# Transcriptomic Meta-Analysis as a Framework for Robust Cross-Study Biological Inference

**DOI:** 10.3390/ijms27114674

**Published:** 2026-05-22

**Authors:** Cinthia Alejandra Olivas-Bernal, Francisco Vargas-Albores, Estefanía Garibay-Valdez, Francesco Cicala, Marcel Martínez-Porchas

**Affiliations:** 1 Centro de Investigación en Alimentación y Desarrollo A.C., Hermosillo 83304, Mexico; colivas125@estudiantes.ciad.mx (C.A.O.-B.); fvalbores@ciad.mx (F.V.-A.); estefania.garibay@ciad.mx (E.G.-V.); 2 Dipartimento di Biomedicina Comparata e Alimentazione, Università degli Studi di Padova, 35020 Padua, Italy; fracicala@gmail.com

**Keywords:** transcriptomic meta-analysis, gene expression integration, batch-effect correction, biological heterogeneity, RNA sequencing

## Abstract

The increasing availability of transcriptomic data has created new opportunities for integrating gene expression studies across biological systems and conditions. However, differences in experimental design, sequencing platforms, and sample composition introduce substantial heterogeneity, limiting direct comparability between studies. Transcriptomic meta-analysis provides a framework to address these challenges by identifying expression patterns that are reproducible across independent datasets. In this review, we outline the key methodological steps involved in transcriptomic meta-analysis, including dataset selection, preprocessing, normalization, batch-effect correction, and statistical integration. We discuss how these steps are influenced by the type of data being analyzed, from microarrays and bulk RNA sequencing to single-cell and spatial transcriptomics. Particular attention is given to the role of technical and biological heterogeneity, which must be explicitly considered to avoid misleading conclusions. Rather than treating heterogeneity solely as a source of noise, we argue that it defines the limits of reproducibility and interpretation in cross-study analyses. By focusing on consistent signals across diverse datasets, transcriptomic meta-analysis enables more robust biological inference and supports applications such as biomarker discovery and disease stratification.

## 1. Introduction

Transcriptomic technologies have become a cornerstone of modern biological and biomedical research, enabling the systematic interrogation of gene expression across tissues, conditions, and organisms. The widespread adoption of high-throughput platforms, initially microarrays (MA), after RNA sequencing (RNA-seq) and later single-cell transcriptomics (SCS), has generated an unprecedented volume of publicly available transcriptomic data, creating new opportunities for secondary analyses and large-scale biological inference [1,2]. Despite this abundance of data, the intrinsic heterogeneity of transcriptomic studies presents a major obstacle to cross-study comparability. Differences in experimental design, sequencing platforms, library preparation protocols, sample composition, and analytical pipelines can introduce non-biological variation that obscures true biological signals [3,4]. As a result, findings from individual transcriptomic studies often exhibit limited reproducibility and context dependence.

Meta-analysis provides a robust statistical framework to overcome limitations of individual transcriptomic studies by integrating results from independent datasets addressing a shared question. By aggregating evidence, it increases statistical power, reduces study-specific noise, and identifies expression patterns that are reproducible across heterogeneous conditions [5,6]. Unlike single-cohort analyses, it emphasizes convergence across studies as a key indicator of biological robustness.

In transcriptomic research, meta-analysis is often confused with mega-analysis. While meta-analysis integrates study-level statistical results obtained independently from each dataset, mega-analysis pools normalized expression data into a single large dataset prior to analysis. Consequently, mega-analysis approaches are generally more dependent on cross-study harmonization procedures to mitigate technical confounding across pooled datasets. Throughout this review, transcriptomic meta-analysis refers specifically to study-level integration approaches. This approach poses specific challenges due to the high dimensionality of the data, complex mean-variance relationships, and susceptibility to technical artifacts such as batch effects. Without rigorous dataset selection, standardized preprocessing, appropriate normalization, and explicit modeling of between-study heterogeneity, analyses may capture technical rather than biological variation [7,8]. When these factors are properly addressed, meta-analysis enables the identification of transcriptional signals consistently observed across independent studies, which often reflect conserved biological programs, such as immune or metabolic response, rather than disease-specific signatures [9,10,11]. Recognizing these trade-offs is essential for the appropriate application of transcriptomic meta-analysis to biomarker discovery, systems biology, and translational research.

Previous methodological reviews have emphasized that transcriptomic meta-analysis requires careful attention to preprocessing, heterogeneity, reproducibility, and statistical modeling choices, particularly when integrating high-dimensional datasets across studies [12,13,14,15]. Building on these methodological foundations, transcriptomic meta-analysis has continued to evolve alongside next-generation sequencing technologies, expanding its scope to RNA-seq, single-cell resolution, and integrative omics analyses.

A common question in transcriptomic meta-analysis concerns the sample size required to obtain reproducible biological signals. In practice, there is no universal threshold, as statistical power depends strongly on the expected effect size and the degree of biological and technical heterogeneity within and across datasets. In highly heterogeneous systems, particularly human tissues such as the brain, reproducible transcriptomic effects often emerge only when analyses include hundreds of samples distributed across multiple cohorts [16,17,18]. By contrast, conditions associated with large transcriptional effects or lower biological variability may yield reproducible signals with substantially smaller sample sizes. Importantly, small transcriptomic studies frequently overestimate effect sizes, a phenomenon consistent with the “Winner’s Curse”, in which initially reported associations tend to diminish as sample size increases [19]. These considerations reinforce that reproducibility in transcriptomic meta-analysis depends less on arbitrary sample thresholds than on the interaction between effect magnitude, heterogeneity structure, and study design.

In practice, transcriptomic meta-analysis has already been applied to biomarker discovery, cross-disease immune profiling, environmental exposure research, and neurodegenerative disorders, illustrating its utility across diverse biological and clinical contexts [20,21,22,23].

This review aims to reframe transcriptomic meta-analysis as a conceptual and methodological framework for extracting biologically meaningful signals from heterogeneous gene expression datasets, emphasizing heterogeneity not merely as a source of noise but as a defining constraint that shapes reproducibility, interpretation, and cross-study integration.

## 2. Fundamentals of Meta-Analysis in Gene Expression Data

Meta-analysis originated as a statistical framework for synthesizing evidence across independent studies addressing a common research question, aiming to increase inferential robustness and explicitly modeling variability across studies [24]. In the context of gene expression research, these principles become more complex because transcriptomic data are indirect measures of cellular functional states rather than primary biological endpoints [25]. Regardless of the underlying technology, microarrays based on probe hybridization, bulk RNA sequencing quantifying mRNA abundance, or single-cell transcriptomic (SCT) approaches resolving expression at cellular resolution, the ultimate goal is to characterize patterns of gene expression that reflect biological function and, ultimately, phenotypic or metabolic outcomes.

From this perspective, transcriptomic meta-analysis extends beyond the integration of heterogeneous sequencing platforms or expression matrices. It seeks to identify reproducible functional signals across studies, tissues, and experimental conditions, while accounting for differences in data structure and biological resolution. Bulk transcriptomic datasets summarize average expression across mixed cell populations, whereas SCT data introduce an additional layer of heterogeneity by capturing cell-to-cell variability and shifts in cellular composition [26]. These differences fundamentally affect variance structure, batch effects, and the definition of comparable units across studies, and therefore require distinct meta-analytic considerations.

Importantly, transcriptomic data do not exist in isolation. Gene expression lies between genomic regulation and downstream molecular phenotypes, including protein abundance and metabolic activity [27]. As a result, transcriptomic meta-analysis increasingly serves as a bridge toward integrative, system-level inference, where reproducible expression patterns are interpreted in the context of proteomic, metabolomic, and other omics datasets. Although the present review focuses on transcriptomic data, many of the methodological principles discussed here are directly relevant to multi-omics integration, as they address shared challenges of heterogeneity, normalization, and cross-study reproducibility.

Accordingly, transcriptomic meta-analysis should be understood as a structured framework for extracting biologically meaningful signals from heterogeneous gene expression datasets. Establishing this conceptual basis is essential before addressing specific data types, preprocessing strategies, and statistical integration methods.

### 2.1. Types of Transcriptomic Data

Transcriptomic technologies have evolved substantially over the past two decades, with each major methodological advance increasing the resolution at which gene expression can be interrogated. Early transcriptomic studies relied primarily on microarray platforms, which quantify gene expression through probe-based hybridization and provide relative expression estimates for predefined transcripts. Although microarrays are limited by probe design, signal saturation, and reduced sensitivity to low-abundance transcripts, they have generated a vast body of publicly available data that remains relevant for integrative analyses. From a meta-analytic perspective, microarray datasets exhibit relatively stable variance structures but are strongly affected by platform-specific effects and differences in probe annotations [28]. Comprehensive discussions of microarray-based meta-analysis can be found elsewhere and are not the focus of the present review [28,29].

The introduction of bulk RNA sequencing (RNA-seq) represented a major shift in transcriptomic analysis by enabling direct quantification of mRNA abundance across the entire transcriptome. Compared with microarrays, RNA-seq can resolve global gene expression patterns and identify novel genes, alternative transcript variants, chimeric transcripts, expressed sequence variants, allele-specific expression, and a wide range of non-coding RNAs, including microRNAs (miRNAs), long non-coding RNAs (lncRNAs), and pseudogenes, even in organisms lacking a reference genome [25,30]. Additionally, microarrays exhibit limited sensitivity for low-abundance transcripts, whereas RNA-seq generally provides improved detection, although its sensitivity remains strongly influenced by sequencing depth and still does not approach the analytical sensitivity of qPCR. However, bulk RNA-seq still provides an averaged expression profile across heterogeneous cell populations within a sample. As a result, observed transcriptional changes may reflect shifts in cellular composition rather than true regulatory changes within specific cell types. This issue becomes particularly relevant in cross-study meta-analyses. On the other hand, RNA-seq generates substantially larger files than microarrays, requiring considerable storage capacity and time-consuming bioinformatic analyses that demand extensive computational resources. Moreover, RNA-seq depends on high-quality RNA samples and careful library preparation to ensure reliable results. Still, RNA-seq remains the superior option for transcriptome analysis and has largely replaced microarrays in most biological and medical research applications [2,5,31].

More recently, single-cell transcriptomic (SCT) technologies have transformed transcriptomics by resolving gene expression at cellular resolution. By capturing cell-to-cell variability, SCT enables the identification of rare cell populations, dynamic cellular states, and context-specific regulatory programs that are invisible in bulk data [32]. At the same time, SCT introduces new analytical challenges for meta-analysis, including increased sparsity, zero inflation, strong batch effects, and variability in cell-type annotation across studies. These features fundamentally alter the unit of integration and require meta-analytic strategies that explicitly account for differences in cellular composition and resolution. Recent computational approaches have attempted to address these limitations through cell-type-aware integration methods, latent-variable modeling, graph-based frameworks, and imputation strategies specifically designed for sparse and zero-inflated transcriptomic data.

Spatial transcriptomics approaches, which couple transcriptomic measurements with spatial coordinates within tissues, introduce an additional layer of complexity. Importantly, spatial transcriptomics does not constitute a distinct sequencing technology but rather an extension of transcriptomic analysis that preserves information about tissue architecture and microenvironmental context [33,34]. By situating gene expression within a spatial framework, these approaches provide critical insight into functional organization, cell–cell interactions, and metabolic gradients. From a meta-analytic perspective, spatial transcriptomics shows that gene expression is not only cell-type-specific but also spatially constrained, reinforcing the need for context-aware integration strategies. However, spatial transcriptomic platforms vary substantially in sequencing depth and sparsity, which may introduce floor effects and limit the detection of low-abundance transcripts across studies.

Together, these technologies generate transcriptomic data with distinct biological resolutions and statistical properties. Understanding their respective strengths and limitations is essential for selecting appropriate meta-analytic frameworks and for interpreting integrated gene expression signals in terms of biological function and metabolic state (Table 1).

While transcriptomic technologies differ in their biological resolution, data structure, and sources of technical variability, these differences alone do not define the meta-analytic strategy. Beyond the choice of platform, transcriptomic meta-analysis critically depends on how gene expression data are summarized, modeled, and integrated across studies. In practice, most transcriptomic meta-analyses can be broadly classified according to the type of analytical question they address: the identification of genes whose expression levels change between conditions, or the characterization of coordinated expression patterns that reflect underlying regulatory or functional networks. These two perspectives correspond to differential expression and co-expression analyses, respectively, and they represent complementary but conceptually distinct frameworks for transcriptomic meta-analysis.

### 2.2. Differential Expression and Co-Expression

Transcriptomic analyses are commonly designed to address one of two broad questions: whether the expression levels of individual genes differ across biological conditions, or whether genes exhibit coordinated expression patterns across samples. Differential expression (DE) analysis focuses on identifying genes whose expression changes significantly between predefined groups [35]. In contrast, co-expression analysis aims to uncover groups of genes that show correlated expression patterns, often interpreted as shared regulatory control or functional association [36,37]. Importantly, the choice between DE and co-expression is not merely methodological but reflects different interpretations of transcriptomic data. Differential expression treats genes as largely independent units of inference, whereas co-expression explicitly models relationships among genes. As a result, DE meta-analyses are generally more sensitive to changes in cellular composition and experimental contrasts. At the same time, co-expression approaches are often more robust to moderate shifts in expression magnitude but remain sensitive to sample size, noise structure, and restricted expression ranges caused by sparsity, low sequencing depth, or overnormalization. Consequently, these approaches address different biological questions and rely on distinct statistical assumptions and sample size requirements.

Differential gene expression analysis typically follows standardized computational pipelines that vary depending on the transcriptomic technology. In RNA-seq, these pipelines commonly include quality assessment of raw reads, preprocessing, alignment or pseudoalignment, expression quantification, and statistical identification of differentially expressed genes [38]. In microarray datasets, preprocessing generally involves background correction, probe summarization, normalization, and probe annotation harmonization prior to differential expression analysis [35].

In contrast, co-expression analysis focuses on identifying relationships among genes within biological networks [39]. In these networks, genes are represented as nodes, and their relationships are edges that reflect correlations in expression patterns across samples. When co-expression patterns change across conditions, this can indicate regulatory alterations affecting the biological processes represented within a given module. The Guilt by Association (GBA) principle assumes that genes with correlated expression are likely to share functional roles [37].

The relevance of this distinction becomes even more pronounced when considering different transcriptomic technologies. In bulk transcriptomic datasets, DE signals may reflect both true regulatory changes and shifts in cell-type proportions. In contrast, co-expression patterns may capture higher-order regulatory programs that persist across conditions. In single-cell transcriptomic data, DE analysis can be performed at the level of individual cell types or states. In contrast, co-expression analyses may reveal cell-type-specific regulatory modules or dynamic transcriptional programs. These differences have direct implications for how results can be meaningfully integrated across studies in a meta-analytic framework.

Together, differential expression and co-expression meta-analyses provide complementary perspectives on transcriptomic data. DE-based approaches are particularly effective for identifying reproducible gene-level signals across heterogeneous datasets, whereas co-expression-based approaches enable the identification of conserved regulatory structures and functional modules. Understanding the strengths and limitations of each strategy is essential for selecting appropriate meta-analytic methods and for interpreting integrated transcriptomic signals in terms of biological function and, ultimately, metabolic and phenotypic outcomes.

### 2.3. Data Sources and Preliminary Selection Criteria

Data acquisition and dataset inclusion represent two closely related yet conceptually distinct steps in transcriptomic meta-analysis. While data sources define the universe of potentially available studies, selection criteria determine whether a given dataset can be meaningfully integrated into a cross-study analytical framework. Separating these processes is essential for understanding how early decisions shape downstream heterogeneity, statistical modeling, and biological interpretation.

#### 2.3.1. Publicly Accessible Transcriptomic Repositories

Public repositories constitute the primary source of transcriptomic data for meta-analytic studies. Databases such as the Gene Expression Omnibus (GEO), the Sequence Read Archive (SRA), and ArrayExpress host a broad spectrum of gene expression datasets generated using microarrays [34,35,36], bulk RNA sequencing, and single-cell transcriptomic technologies across diverse biological conditions and experimental designs. The widespread adoption of data-sharing policies has led to a vast, continually expanding body of publicly accessible transcriptomic data.

The availability of raw or minimally processed data within these repositories is a critical prerequisite for transcriptomic meta-analysis, as it enables reprocessing under standardized analytical pipelines. However, data availability alone does not guarantee suitability for integration. Public repositories differ in metadata completeness, annotation consistency, and reporting quality, and these differences can substantially limit cross-study comparability. While these repositories provide access to a wide range of transcriptomic datasets, their suitability for meta-analysis ultimately depends on adherence to transparent selection criteria and FAIR data principles [37].

Consequently, the role of public databases in transcriptomic meta-analysis is defined not only by data volume, but by the extent to which deposited datasets support transparent reinterpretation and methodological harmonization. In this context, publicly accessible repositories, unlike controlled-access or proprietary resources, enable independent reprocessing and large-scale data reuse, both of which are essential for reproducibility and integrative analyses. However, maximizing their analytical value requires careful dataset curation, including the application of explicit selection criteria and rigorous quality assessment to ensure cross-study comparability.

The major public repositories commonly used for transcriptomic data reuse and integrative analyses, together with their key characteristics, strengths, and limitations from a meta-analytic perspective, are summarized in Table 2. Beyond conventional bulk and single-cell datasets, emerging resources such as SpatialDB (http://www.spatialomics.org/SpatialDB/ accessed on 18 May 2026), which provides information on spatially resolved transcriptomes [38], and STOmicsDB (https://db.cngb.org/stomics/ accessed on 18 May 2026), which supports the analysis, visualization, and comparison of spatial transcriptomic datasets [39], further expand the scope of publicly available transcriptomic data. These developments increase both the opportunities for integrative analyses and the methodological complexity associated with incorporating spatial context into transcriptomic meta-analysis.

#### 2.3.2. Dataset Selection and Retrieval Strategies

The inclusion of datasets in transcriptomic meta-analysis requires the application of explicit and transparent selection criteria to ensure both biological relevance and analytical feasibility. In practice, dataset selection is commonly guided by standardized reporting frameworks and data-sharing principles, most notably the Preferred Reporting Items for Systematic Reviews and Meta-Analyses (PRISMA) guidelines [40] and the FAIR data principles [41].

A comprehensive study identification process typically involves searching across multiple literature databases, as individual repositories index partially overlapping sets of studies and journals [42]. In addition to graphical web interfaces, several transcriptomic repositories support formalized search syntax and application programming interfaces (APIs), enabling automated and reproducible dataset retrieval. In particular, tools such as the GEOquery package for Bioconductor facilitate direct interaction with GEO and related repositories, improving both search transparency and workflow reproducibility [43].

Robust search strategies combine controlled vocabularies (e.g., MeSH or EMTREE terms) with free-text keywords, synonyms, spelling variants, Boolean operators, and study-design filters to balance precision and coverage. Controlled vocabularies enhance retrieval consistency by mapping query terms to standardized biological concepts, thereby reducing ambiguity across heterogeneous naming conventions. At this stage, searches are generally restricted to original, peer-reviewed transcriptomic studies to ensure analytical relevance [44].

Within this framework, PRISMA guidelines are particularly useful for formalizing inclusion and exclusion criteria based on biological comparability, including species, tissue or cell type, experimental condition, and study design, as well as for documenting decision points throughout the dataset selection process [45,46]. Interactive versions of the PRISMA 2020 checklist are also publicly available and may facilitate standardized reporting and study documentation. Although the information required for transcriptomic meta-analysis should, in principle, be available in the metadata associated with each dataset, a major limitation of publicly accessible omics repositories is the frequent absence or incompleteness of dataset annotations, despite metadata submission being a formal requirement.

Several initiatives have sought to mitigate this limitation, including the development of standardized metadata guidelines such as the FAIR Data Principles [46]. Nevertheless, missing or poorly annotated metadata remains a pervasive challenge. In practice, this often forces researchers to contact original study authors to obtain essential experimental or sample-level information, a process that is time-consuming and frequently unsuccessful.

Importantly, dataset selection is not a purely logistical step but a foundational methodological decision. Inconsistencies in experimental design, incomplete annotation, or ambiguous group definitions introduce sources of heterogeneity that cannot be fully mitigated through downstream normalization or batch-effect correction. Consequently, decisions made at this stage directly constrain subsequent analytical strategies and the scope of biological interpretation.

Together, PRISMA-guided study selection and adherence to FAIR data principles define the boundaries within which transcriptomic meta-analyses operate, establishing a methodological foundation for robust and interpretable cross-study integration.

### 2.4. Heterogeneity and Integration Challenges

Transcriptomic meta-analysis operates under the fundamental constraint that gene expression datasets generated across independent studies are inherently heterogeneous. This heterogeneity arises not only from technical differences in data generation and processing, but also from biological variability linked to study design, population structure, tissue composition, and analytical resolution. Unlike single-study analyses, cross-study integration requires explicitly acknowledging and modeling these sources of variability, as they directly shape the reliability and interpretability of meta-analytic inference. Understanding the nature and origin of heterogeneity is therefore a prerequisite for selecting appropriate normalization, batch-correction, and statistical integration strategies, which are addressed in the following sections.

#### 2.4.1. Batch Effect and Technique Variability

Technical heterogeneity constitutes one of the most pervasive challenges in transcriptomic meta-analysis. It arises from systematic non-biological differences introduced during data generation and processing, including variability in sequencing platforms, library preparation protocols, reagent batches, laboratory environments, and computational preprocessing pipelines [47]. Because transcriptomic meta-analyses integrate data across studies, these technical differences often align with study identity, making them particularly difficult to disentangle from biological effects.

These non-biological experimental variations, also known as batch effects [8], represent a prominent manifestation of technical heterogeneity. Importantly, they do not simply introduce random noise but can induce structured biases that alter gene expression distributions in a study-specific manner. When unaddressed, batch effects inflate between-study variability, distort effect-size estimates, and reduce statistical power, obscuring the true biological signals and ultimately compromising the reproducibility of meta-analytic findings [4,48]. The “omics” field is particularly vulnerable to these issues because it integrates multiple data types characterized by distinct distributions, dynamic ranges, and measurement scales, often generated across heterogeneous analytical platforms.

The impact of technical heterogeneity is amplified when integrating datasets generated using different transcriptomic technologies or analytical workflows. Differences between microarray and RNA-seq platforms, as well as variability in sequencing depth, alignment strategies, and quantification methods, further contribute to non-biological variation. In single-cell transcriptomic data, technical heterogeneity is often exacerbated by strong batch effects arising from cell capture, library construction, and sequencing chemistry, which can dominate biological signals if not carefully accounted for [49].

Recognizing technical heterogeneity and batch effects as structured sources of variation, rather than incidental artifacts, is essential for the design of transcriptomic meta-analyses. This recognition motivates the use of explicit normalization, batch-effect correction, and statistical modeling strategies, which are discussed in detail in the subsequent methodological section.

The increasing reuse of transcriptomic data from public repositories has amplified the practical relevance of batch effects in integrative analyses. In this context, minimizing technical bias requires not only appropriate correction procedures but also the consistent application of comparable analytical pipelines and, whenever possible, the use of raw rather than previously processed data [3,4,8,46].

#### 2.4.2. Biological Heterogeneity and Study Design

In addition to technical variability, biological heterogeneity represents a major challenge for transcriptomic meta-analysis. Differences in population characteristics, disease stage, treatment exposure, tissue sampling, cellular composition, and experimental design introduce genuine biological variability that is intrinsic to the systems under study [9]. Unlike technical heterogeneity, biological variation is not an artifact to be removed, but a defining feature that must be carefully interpreted within a meta-analytic framework.

In bulk transcriptomic studies, biological heterogeneity is often compounded by variation in cellular composition across samples. Changes in gene expression detected at the tissue level may reflect shifts in the relative abundance of specific cell types rather than regulatory changes occurring within individual cells [50,51]. When integrating bulk datasets across studies, such compositional effects can generate apparent inconsistencies in differential expression signals, even when underlying cell-type-specific programs are conserved.

Study design choices further shape biological heterogeneity in transcriptomic meta-analysis. Variability in inclusion criteria, control definitions, sampling strategies, and experimental contrasts can lead to differences in baseline expression profiles and effect sizes across studies. These factors contribute to between-study variability that cannot be fully reconciled through statistical adjustment alone and must instead be considered during study selection and interpretation.

Single-cell transcriptomic data add a further layer of biological heterogeneity because differences in cell-type definitions, annotation strategies, and cellular-state composition may vary substantially across studies [1,32]. Consequently, integrating single-cell datasets or combining single-cell and bulk data requires explicit consideration of how biological variability is represented at different levels of resolution. Recent studies have further shown that meta-analytic integration can substantially improve the reproducibility of differentially expressed genes in single-cell studies of highly heterogeneous disorders such as neurodegenerative diseases [52].

Additionally, the sequencing platforms chosen in each study can complicate meta-analytic integration. Studies relying on short-read technologies (e.g., Illumina) are typically optimized for gene-level quantification. In contrast, those employing long-read platforms, such as Oxford Nanopore or Pacific Biosciences (PacBio) SMRT sequencing, are better suited for characterizing isoforms, splice variants, and epigenetic modifications. These methodological discrepancies, including differences in read length, platform chemistry, and experimental objectives, can generate inconsistencies that further complicate cross-study integration [53].

From a meta-analytic perspective, distinguishing meaningful biological heterogeneity from confounding variation is a central conceptual challenge. Overly aggressive correction strategies risk removing biologically relevant signals, whereas insufficient control may preserve context-specific effects that limit generalizability. Recognizing the biological origins of heterogeneity is therefore essential for interpreting transcriptomic meta-analysis results and for selecting appropriate integration strategies, as discussed in the following sections.

#### 2.4.3. Resolution-Dependent Challenges

Transcriptomic meta-analysis is further complicated by differences in biological resolution across studies. Bulk and single-cell transcriptomic approaches capture gene expression at fundamentally different levels of organization, producing distinct data structures, variance profiles, and interpretive frameworks. As a result, heterogeneity in transcriptomic meta-analysis is not only study-dependent but also resolution-dependent.

Bulk and single-cell datasets differ not only in scale but also in the very unit of biological interpretation. Bulk data aggregate signals across mixed cell populations, whereas single-cell data resolve expression at the level of individual cells [32]. These differences affect variance structure, sparsity, batch sensitivity, annotation consistency, and the comparability of inferred biological signals across studies.

Integrating transcriptomic data across resolutions or combining bulk and single-cell datasets within a unified meta-analytic framework poses a distinct conceptual challenge. While single-cell data can inform the interpretation of bulk expression patterns by clarifying cell-type contributions, differences in resolution, scale, and variance structure limit direct comparability. Without careful consideration, such integration risks conflating biological signal with resolution-driven artifacts.

Resolution-dependent differences, therefore, shape not only the structure of transcriptomic data but also the assumptions required for valid cross-study integration. Recognizing these constraints is essential for selecting appropriate harmonization and meta-analytic strategies.

## 3. Methodology for Transcriptomic Meta-Analysis

**Figure 1:**
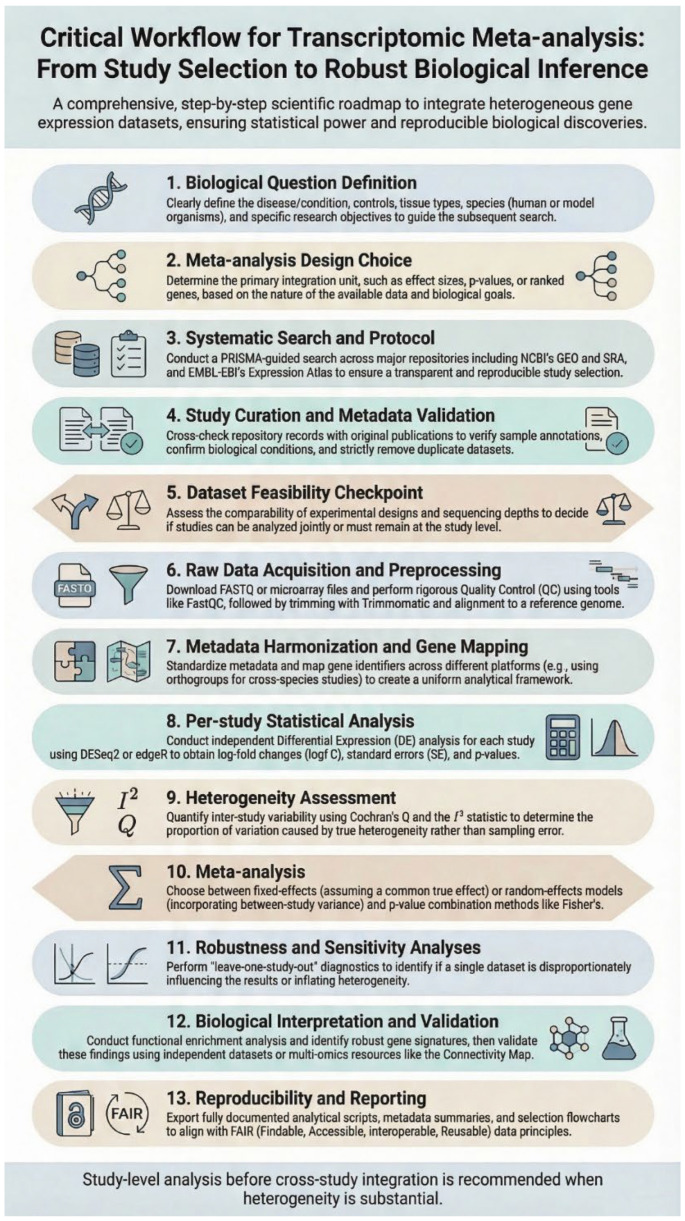
Critical workflow for transcriptomic meta-analysis. The figure summarizes the main step-by-step process of transcriptomic meta-analysis, beginning with biological question definition and meta-analytic design selection, followed by systematic dataset retrieval, study curation, metadata validation, and feasibility assessment. It then proceeds through raw data acquisition, preprocessing, metadata harmonization, per-study statistical analysis, heterogeneity assessment, meta-analysis, robustness testing, biological interpretation, validation, and reproducible reporting.

Transcriptomic meta-analysis requires a rigorous, carefully structured analytical workflow to preserve biological signals while appropriately controlling technical and study-specific variability (Figure 1). As discussed in the preceding sections, heterogeneity arising from experimental design, data generation protocols, and biological resolution imposes fundamental constraints on cross-study integration. Traditional approaches such as global normalization, principal component analysis-based adjustment, or machine learning classifiers (e.g., support vector machines) have been used to mitigate inter-study variability. More recently, machine learning and artificial intelligence approaches have also been incorporated into transcriptomic integration frameworks for dimensionality reduction, feature selection, latent-pattern identification, and predictive modeling across highly heterogeneous datasets [54]. However, these strategies often perform poorly when the number of samples per study is small or when multiple batches are present, a common scenario in transcriptomic meta-analyses. Moreover, several of these methods assume normally distributed input data, which is inconsistent with the discrete and overdispersed nature of RNA-seq read counts [7].

Therefore, the methodological decisions involved in preprocessing, normalization, batch-effect correction, and statistical integration are crucial for ensuring the robustness, reproducibility, and interpretability of meta-analytic findings. Several of the core principles discussed here—such as effect-size integration, *p*-value aggregation, and rank-based methods—have been systematically reviewed in the context of gene expression meta-analysis [12], (A comparative summary of major statistical approaches commonly used in transcriptomic meta-analysis is provided in Table 3). This section highlights the fundamental methodological aspects of transcriptomic meta-analysis, focusing on core principles and strategies rather than particular software tools.

### 3.1. Data Preprocessing and Normalization

Transcriptomic meta-analysis typically begins with dataset-level preprocessing to ensure data quality and comparability before integration (Figure 1). When raw data are available, this step includes quality assessment of sequencing reads or probe-level signals, removal of low-quality samples, and filtering of low-abundance or low-information features, commonly using FastQC and Trimmomatic. Although these procedures are often standardized within individual studies, their consistent application across datasets is essential in a meta-analytic context to minimize downstream technical variability [7].

Several large-scale initiatives have further sought to standardize preprocessing across publicly available transcriptomic datasets through uniform quality control, re-alignment, normalization, and annotation pipelines. Resources such as Gemma, ARCHS4, and DEE2 provide uniformly processed transcriptomic data that can facilitate cross-study integration and improve reproducibility in meta-analytic workflows [55,56,57]. Additional computational tools commonly used for transcriptomic meta-analysis, preprocessing, batch correction, and integrative workflows are summarized in Table 4.

Normalization is a critical step in transcriptomic data integration, correcting for systematic differences in signal intensity or sequencing depth while preserving biologically meaningful variation. Major normalization and batch-correction methods relevant to transcriptomic meta-analysis are summarized in Table 5. In bulk transcriptomic data, normalization methods are typically designed to account for differences in library size, composition bias, or probe intensity distributions [58,59]. However, the assumptions underlying these methods, such as the expectation that most genes are not differentially expressed, may not hold uniformly across heterogeneous datasets, particularly those from different platforms or experimental conditions. Furthermore, normalization and harmonization strategies differ substantially in their computational assumptions, scalability, and robustness across transcriptomic contexts. Methods optimized for bulk RNA-seq integration may perform poorly in highly sparse single-cell datasets, whereas aggressive harmonization procedures may obscure biologically meaningful heterogeneity in study-level meta-analysis designs.

In analytical workflows restricted to RNA-seq data derived from a single study, commonly applied normalization procedures include Reads Per Kilobase of transcript per Million mapped reads (RPKM) and the Trimmed Mean of M-values (TMM) [60]. However, the challenge of normalization is further amplified in multi-study settings. Methods that perform adequately within a single experiment may introduce bias when applied across studies with distinct variance structures or expression distributions. Consequently, normalization in transcriptomic meta-analysis must be evaluated not only for within-study performance but also for its impact on cross-study comparability and downstream heterogeneity. Methods such as the Trimmed Mean of M-values (TMM), implemented in edgeR [61,62], and the Median of Ratios, implemented in DESeq2 [63,64], are widely regarded as superior to other strategies. These approaches are particularly effective because they assume most genes are not differentially expressed, allowing them to adjust for compositional differences between samples and ensure that downstream analyses reflect true biological convergence rather than technical artifacts [61,63,64,65].

In integrative analyses combining microarray and RNA-seq data, limma-based frameworks have proven particularly useful due to their flexibility and robust performance across transcriptomic platforms. In this context, the voom transformation enables RNA-seq count data to be modeled using linear modeling strategies originally developed for microarrays, facilitating more consistent cross-platform analyses [66,67].

In cross-species transcriptomic meta-analysis, an additional challenge involves the harmonization of orthologous genes across organisms. Because gene identifiers and evolutionary relationships are not always directly comparable between species, orthology mapping becomes essential for identifying conserved transcriptional programs. Several resources are commonly used for this purpose, including HCOP (HGNC Comparison of Orthology Predictions), OrthoDB, and BioMart/biomaRt, which facilitate the retrieval, annotation, and integration of orthologous genes across multiple species. These tools are particularly valuable in comparative and evolutionary transcriptomics, where biological interpretation depends on distinguishing conserved functional signals from species-specific expression patterns.

In single-cell transcriptomic data, preprocessing and normalization pose additional challenges due to data sparsity, zero inflation, and strong technical effects associated with cell capture and sequencing depth [68,69]. While single-cell-specific normalization frameworks have been developed, their application in meta-analytic contexts requires careful consideration of how normalization interacts with batch effects, cell-type composition, and study-specific resolution [70,71].

Taken together, preprocessing and normalization are not merely preparatory steps but foundational methodological decisions in transcriptomic meta-analysis. Their selection directly influences the effectiveness of subsequent batch-effect correction and statistical integration strategies, which are discussed in the following subsections.

### 3.2. Batch-Effect Correction and Cross-Study Harmonization

Batch-effect correction represents a central step in transcriptomic meta-analysis, as it addresses structured sources of technical variability that arise when integrating datasets across independent studies. As discussed in Section 2.4, batch effects arise from systematic non-biological differences across experimental protocols, sequencing platforms, laboratory environments, and computational processing pipelines. In multi-study analyses, these effects frequently align with study identity, making them particularly detrimental to cross-study inference if left unaddressed. The use of uniformly reprocessed public datasets may partially mitigate some of these sources of technical variability by reducing preprocessing inconsistencies across studies.

Unlike normalization, which primarily adjusts within-sample expression distributions, batch-effect correction aims to remove study-specific biases while preserving biological variation in interest [72]. This distinction is critical in transcriptomic meta-analysis, where overcorrection may eliminate genuine biological signals, whereas insufficient correction may retain confounding technical effects that inflate heterogeneity between studies.

Several statistical frameworks have been developed to address batch effects in transcriptomic data. Linear modeling approaches, such as empirical Bayes-based methods, estimate and adjust batch-associated effects across genes, assuming that technical variation can be modeled as an additive or multiplicative component [72]. In the context of count-based RNA-seq data, extensions of these frameworks account for the discrete nature and mean-variance relationship of expression counts [73]. These approaches have been widely adopted due to their flexibility and scalability in multi-study settings.

Importantly, the necessity and appropriateness of batch-effect correction depend strongly on the meta-analytic framework being applied. In mega-analysis approaches, where normalized expression matrices from multiple studies are pooled into a single dataset prior to statistical analysis, cross-study harmonization is often essential to reduce confounding introduced by technical variability, platform differences, sequencing depth, and study-specific mean-variance structures. In contrast, traditional transcriptomic meta-analysis approaches based on independent per-study differential expression analyses followed by effect-size or *p*-value integration generally require less aggressive cross-study correction, since statistical integration occurs at the study level rather than directly on pooled expression matrices. In these contexts, excessive harmonization may inadvertently remove biologically meaningful between-study heterogeneity, particularly when datasets differ substantially in experimental design, population structure, or sample composition.

Batch-effect correction becomes more complex when technical and biological factors are confounded, such as when all samples from a given condition originate from a single study. In such cases, separating technical variation from biological signal may be statistically infeasible, and corrective procedures risk distorting the true underlying effects. Consequently, batch-effect correction strategies must be informed by study design considerations and applied in conjunction with careful dataset selection and sensitivity analyses.

When integrating expression data from both microarray and RNA-seq platforms, cross-platform harmonization strategies are generally classified into two groups. Joint normalization methods, including Quantile Normalization (QN), ComBat, RNABC, Shambhala2, and Angel’s method, simultaneously align the distributions of multiple datasets. In contrast, separate normalization techniques treat each dataset independently, such as Training Distribution Matching (TDM) and MatchMixer, the latter specifically designed to enable cross-platform [58]. A more user-friendly alternative applicable to both RNA-seq and microarray data is z-score normalization, which effectively reduces technical and systematic variation [59]. Despite these advancements, the overall sensitivity for detecting differentially expressed genes across platforms remains low, regardless of the strategy employed [58].

In single-cell transcriptomic datasets, batch effects are often more pronounced due to variations in cell capture efficiency, library preparation, and sequencing depth [74]; see also recent discussions on overcorrection in scRNA-seq integration). Correction methods must account for variability both at the individual cell level and across entire studies. Although several batch-correction techniques tailored for single-cell data exist, their application in meta-analytic settings requires caution, as overly aggressive alignment of cellular manifolds might obscure significant biological differences between studies.

Overall, batch-effect correction should be viewed not as a purely technical step but as a modeling decision that directly affects heterogeneity estimates and downstream statistical integration. Its role is therefore closely tied to the choice of meta-analytic framework, as discussed below.

#### Batch-Effect Correction and Experimental Biases

To address the limitations of Gaussian-based correction methods in count-based transcriptomic data, ComBat-seq has emerged as a robust alternative specifically designed for RNA-seq datasets. Unlike the original ComBat algorithm, ComBat-seq models gene expression using a negative binomial distribution, thereby preserving the integer structure and mean-variance relationship inherent to RNA-seq data [7,73]. Recent implementations such as pyComBat have further reduced computational cost while preserving the empirical Bayes framework, facilitating the routine application of correction procedures in large-scale integrative analyses [75,76].

Nevertheless, these approaches still rely on assumptions about data distribution and the relationship between biological conditions and batch structure. Their performance may deteriorate when technical and biological factors are strongly confounded, as when all samples from a given condition originate from a single study. Under these circumstances, separating technical from biological variation may be statistically infeasible, and correction procedures risk distorting the underlying signal. From a meta-analytic perspective, this limitation is especially important because unresolved batch structure can inflate between-study heterogeneity, bias model selection, and promote spurious differential expression. Batch correction should therefore not be interpreted as an automatic preprocessing step universally applicable to all transcriptomic integration strategies. Rather, its implementation must remain closely linked to study design, the degree of technical confounding, and the specific meta-analytic framework employed. While aggressive harmonization may improve comparability in pooled mega-analysis designs, it may also obscure biologically meaningful heterogeneity in study-level meta-analytic approaches.

### 3.3. Strategies for Transcriptomic Meta-Analysis

Once transcriptomic datasets have been preprocessed, normalized, and harmonized, the core task of meta-analysis requires statistical frameworks capable of simultaneously capturing biological convergence and quantifying inter-study variability. Broadly, transcriptomic meta-analytic strategies can be classified into two categories: those that integrate effect sizes directly and those that aggregate measures of statistical significance. These approaches are complementary rather than competing, and their suitability depends on dataset comparability, heterogeneity structure, and the biological question under investigation.

#### 3.3.1. Effect-Size-Based Models

The choice between fixed- and random-effects models represents a fundamental decision in meta-analysis because it defines how biological generalization is interpreted. In the fixed-effects framework, all studies are assumed to estimate the same underlying true effect size, and the observed differences among them are attributed exclusively to sampling error. Under this assumption, studies with larger sample sizes receive greater weight because their variance is lower, resulting in more precise combined estimates [77].

In contrast, the random-effects model assumes that effect sizes vary across studies because each experiment reflects a different realization of the biological process. Here, the observed variance is partitioned into two components: within-study variance and between-study variance. This additional variance term leads to wider confidence intervals but provides a more realistic representation of transcriptomic data integration, where differences in experimental design, tissue source, sequencing platform, and population background are unavoidable [24].

The evaluation of heterogeneity is therefore a central step in model selection. Statistics such as Cochran’s Q and Higgins’ I^2^ are commonly used to quantify variability between studies. However, both have important limitations. Cochran’s Q has low statistical power when the number of studies is small, but it becomes overly sensitive in large meta-analyses. Higgins’ I^2^, in contrast, expresses heterogeneity as a relative proportion of total variability and does not indicate the magnitude of the absolute variance [78]. In transcriptomic meta-analysis, where methodological diversity is the rule rather than the exception, the random-effects model is generally more appropriate because it allows extrapolation beyond the specific datasets included in the analysis and enables more robust datasets to be constructed.

However, this preference should not be interpreted as universal. Fixed-effects models remain valid when the objective is to estimate a common effect within a highly controlled, methodologically homogeneous dataset. Importantly, applying a fixed-effects model to heterogeneous data leads to overconfident estimates, whereas using a random-effects model in a homogeneous context primarily reduces statistical precision without compromising validity.

#### 3.3.2. *p*-Value-Based Integration Methods

*p*-value-based methods provide an alternative approach for transcriptomic meta-analysis by combining statistical evidence rather than relying on quantitative effect estimates. Techniques such as Fisher’s and Stouffer’s methods analyze each dataset independently, then aggregate the resulting *p*-values to pinpoint genes with consistent differential expression across studies [29,79]. Comparative evaluations of these approaches in transcriptomic datasets have shown that their performance may vary substantially depending on heterogeneity structure, study size, and effect distribution [14,15,80]. These methods are especially valuable when effect sizes are difficult to compare due to differences in analytical pipelines or platform-specific scaling. By emphasizing statistical significance, *p*-value-based strategies reduce sensitivity to variations in effect magnitude between studies while preserving information about the reproducibility of gene-level signals.

Fisher’s method for combining probabilities converts individual *p*-values into a chi-square statistic, with larger values indicating stronger evidence against the null hypothesis across studies. This method is used in tools like metaRNASeq and is commonly used to identify differentially expressed genes, often with false discovery rate (FDR) control to account for multiple testing. Stouffer’s Z-method offers an alternative approach in which *p*-values are transformed into standardized Z-scores and then combined, allowing the use of study-specific weights. Since Z-scores place gene expression data on a common scale, this technique is especially suitable for integrating transcriptomic data from different platforms [59]. Conversely, Edgington’s method directly sums *p*-values and is less affected by extremely small values, which can be useful when signals vary in strength [81].

A limitation of *p*-value-based methods is their limited ability to preserve effect directionality, which can complicate biological interpretation and increase sensitivity to recurrent confounding signals or technical artifacts shared across studies. To partially address this limitation, some transcriptomic meta-analytic frameworks incorporate directional information by assigning the sign of the effect to transformed *p*-values or by using directional vote-counting strategies that quantify the consistency of up- or downregulation across studies [82,83]. Furthermore, many traditional methods assume independence among studies, an assumption that can be violated in transcriptomic research when datasets share samples, platforms, or preprocessing procedures. In such cases, methods based on the Cauchy combination test offer a more suitable approach because they explicitly incorporate correlation among *p*-values, thereby minimizing false-positive inflation [58].

From a biological perspective, *p*-value aggregation detects genes that reliably respond across various experiments, while effect-size models measure the strength of that response. For biomarker discovery, the most effective approach typically combines both methods: first correcting for technical biases and heterogeneity, and then identifying consistent transcriptional signals through integrated statistical evidence.

### 3.4. Network-Based and Co-Expression Meta-Analysis Approaches

Beyond gene-level differential expression, transcriptomic meta-analysis can be extended to integrate co-expression patterns and regulatory networks [84]. Co-expression meta-analyses aim to identify gene modules or network structures that are reproducible across studies, rather than focusing on individual genes [85].

Network-based approaches are particularly valuable for capturing higher-order biological organization and for reducing sensitivity to study-specific noise. Modules defined by correlated expression patterns often correspond to shared regulatory mechanisms or functional pathways and may be more stable across heterogeneous datasets than single-gene signals.

By leveraging gene co-expression networks, specifically through the Weighted Gene Co-expression Network Analysis (WGCNA), it is possible to identify highly preserved gene modules that share functional commonalities across diverse cohorts. These methods prioritize “hub genes” based on their connectivity, providing a more stable biological signal than traditional differential expression analysis and ensuring that identified biomarkers are consistent across different experimental conditions [86,87].

Furthermore, network-centric approaches allow for the projection of multi-study data onto established biological interactomes to perform de novo network enrichment. Utilizing algorithms such as score propagation or differential co-expression analysis, researchers can detect the “rewiring” of molecular interactions that characterize complex disease states [88,89]. This systematic synthesis of data facilitates the reconstruction of Gene Regulatory Networks (GRNs), moving beyond static gene lists to reveal the dynamic, modular architecture of the transcriptome. Such integrative methodologies are essential for pinpointing master regulators and pathways with high potential for clinical intervention.

Although co-expression meta-analysis is computationally more complex than gene-level integration, it provides a complementary perspective that aligns naturally with systems biology and functional interpretation. As such, network-based strategies play an increasingly important role in transcriptomic meta-analysis, particularly for studies focused on pathways, cellular states, and metabolic regulation.

### 3.5. Computational Frameworks and Reproducibility

The practical use of transcriptomic meta-analysis depends heavily on R, the most popular computational environment in bioinformatics and statistical genomics. R offers a flexible, extendable platform for data handling, statistical modeling, and visualization, making it ideal for integrative transcriptomic studies that demand transparency and reproducibility across complex workflows.

Within this ecosystem, Bioconductor is a cornerstone of transcriptomic data analysis, providing a curated collection of open-source R packages for high-throughput biological data analysis. In contrast, the Comprehensive R Archive Network (CRAN) serves as the primary repository for general-purpose R packages, including tools for statistical modeling, data integration, and workflow management [90,91]. Together, R, Bioconductor, and CRAN constitute the most widely used computational framework for transcriptomic meta-analysis, providing direct access to methods for differential expression analysis, batch-effect correction, statistical integration, and downstream functional interpretation in a modular, interoperable environment.

Beyond specific software tools, effective transcriptomic meta-analytic workflows are characterized by common methodological principles rather than particular algorithms. These include consistent preprocessing and normalization procedures, transparent decision-making in analysis, and clear documentation of assumptions and parameter selections. Reproducibility is especially vital in transcriptomic meta-analysis, given the need for cumulative evidence synthesis and the reliance on data reuse across multiple studies.

Standardized analytical pipelines, version-controlled code, and fully documented workflows are therefore essential for enabling independent validation and reuse of meta-analytic results. Analytical decisions at each stage of the pipeline, from dataset selection and preprocessing to statistical integration and biological interpretation, should be explicitly reported to ensure interpretability, reproducibility, and long-term usability of transcriptomic meta-analysis studies. From a practical perspective, workflow automation, containerized computational environments, benchmark datasets, and explicit reproducibility metrics may further strengthen the transparency, portability, and long-term reusability of transcriptomic meta-analysis pipelines.

## 4. Applications of Transcriptomic Meta-Analysis

The practical importance of transcriptomic meta-analysis becomes evident when statistical integration produces biologically meaningful and clinically useful results. Its main benefit lies not only in enhancing statistical power but also in filtering transcriptional signals through cross-study reproducibility. Genes that remain significant across diverse experimental conditions are more likely to reflect fundamental disease mechanisms rather than cohort-specific artifacts, making them stronger candidates for biomarker development. This focus on reproducibility shifts biomarker discovery from a context-dependent observation to a convergence-based inference, a crucial step for translational applications in varied clinical environments [6,28].

Transcriptomic meta-analysis has also attracted considerable interest in translational applications such as drug repurposing, where disease-associated expression signatures are compared against transcriptional responses induced by pharmacological compounds. However, the interpretability of these approaches depends strongly on the quality, reproducibility, and biological relevance of the reference transcriptional datasets used to construct drug-response databases. Many of these resources are derived from small-scale experiments, frequently performed under highly controlled in vitro conditions, which may limit their generalizability across biological systems and clinical contexts. Consequently, although transcriptomic signature matching remains a promising strategy for hypothesis generation, repurposing predictions should be interpreted cautiously and ideally supported by independent experimental or clinical validation.

Nevertheless, this same convergence principle introduces an inherent analytical tension: meta-analysis tends to favor transcriptional programs shared across studies, often linked to fundamental biological processes such as inflammation, cell-cycle regulation, or metabolic stress [11,92]. While this increases robustness, it may reduce disease specificity if functional context is not incorporated into downstream interpretation. Consequently, biomarker discovery in a meta-analytic framework should be understood not as the identification of universally altered genes but as the detection of transcriptional patterns whose reproducibility exceeds the variability introduced by experimental design, population structure, and sequencing technology.

### 4.1. Biomarker Discovery and Gene Signatures

The persistence of differential expression across heterogeneous datasets, even after a strict pipeline that accounts for quality, denoising, and normalization, indicates that the observed transcriptional changes are not driven by specific sampling protocols or technical configurations. Rather than reflecting the magnitude of the effect in a single cohort, this consistency suggests that the signal’s direction and structure remain stable despite variations in population stratification, sequencing platforms, and analytical pipelines. Such stability is particularly relevant for distinguishing biologically structured responses from those that arise from study design, thereby strengthening the interpretive coherence of cross-study comparisons.

The meta-analytic strategy typically involves conducting independent differential expression analyses followed by integrative statistical modeling. This approach preserves within-study biological structures while enabling detection of genes that show coordinated responses across experiments. Importantly, the resulting candidates are not simply differentially expressed but are meta-significant, meaning their statistical support derives from multiple independent observations [6]. This distinction is critical because clinical biomarkers must operate under conditions of biological and technical heterogeneity rather than within the controlled environment of a single experiment.

Nevertheless, the statistical recurrence that defines meta-analytic biomarkers does not necessarily imply mechanistic relevance. In practice, highly reproducible genes often reflect shifts in cell composition or conserved stress responses rather than disease-initiating events [93,94]. Therefore, integrating functional enrichment, tissue specificity, and, when available, single-cell resolution is essential for distinguishing causal candidates from transcriptional correlates.

#### Identification of Disease Biomarkers Through Transcriptomic Meta-Analysis

A paradigmatic example of the capacity of transcriptomic meta-analysis to generate clinically relevant biomarkers is the identification of gene-expression predictors of tamoxifen response in breast cancer. By integrating multiple independent cohorts, Mihály et al. [23] demonstrated that the expression levels of PGR (progesterone receptor), MAPT (microtubule-associated protein tau), and SLC7A5 (large neutral amino-acid transporter 1) constitute a robust predictive signature of therapeutic outcome. These genes did not show uniform significance across all individual datasets; rather, their relevance emerged from their consistent behavior across studies. This illustrates a central property of meta-analysis: it rescues biologically meaningful signals that remain below the detection threshold in underpowered or heterogeneous individual cohorts.

In the context of environmental exposure, a large-scale meta-analysis of whole-blood transcriptomes identified a reproducible gene expression signature associated with cigarette smoking [21]. The study revealed coordinated dysregulation of genes involved in xenobiotic metabolism, immune response, and oxidative stress across multiple population-based cohorts. The signature’s robustness across demographic and technical variability not only supports its use as an exposure biomarker but also establishes a mechanistic link to smoking-related diseases. At the same time, the recurrence of immune-related genes across unrelated pathological conditions highlights a recurring challenge in meta-analytic biomarker discovery: highly reproducible transcriptional signals may reflect shared systemic responses rather than disease-specific molecular events.

In neurodegenerative disorders, integrating multiple brain transcriptomic datasets enabled the identification of Alzheimer’s disease-associated transcriptional alterations that were not detectable in individual studies because of regional heterogeneity and limited sample size [22]. The meta-analysis revealed convergent dysregulation of immune and synaptic pathways, enabling the prioritization of candidate therapeutic targets. Importantly, the study demonstrated that meta-analytic integration can disentangle disease-associated signals from the intrinsic transcriptional variability of anatomically and functionally distinct brain regions, a major limitation in single-cohort analyses.

Similarly, cross-disease transcriptomic integration has revealed conserved pathogenic immune-cell programs. The expansion of CXCL10^+^ CCL2^+^ macrophage signatures across severe COVID-19 and other inflammatory conditions illustrates how meta-analysis can redefine biomarker discovery by identifying molecular phenotypes that transcend traditional clinical classifications [20]. While this convergence increases biological robustness, it also suggests that these signatures may be better biomarkers of pathological processes (e.g., hyperinflammation) than disease-specific diagnostic tools.

From a methodological perspective, these examples also show that the statistical framework used for integration influences which biomarkers are identified. Effect-size models tend to prioritize genes with large, consistent expression changes, whereas *p*-value aggregation methods detect genes with reproducible but potentially moderate effects. In practice, combining both approaches often yields robust, biologically interpretable signatures.

Ultimately, transcriptomic meta-analysis reframes biomarker discovery as a reproducibility challenge across biological and technical dimensions. Its most significant contribution is not generating longer gene lists but identifying transcriptional programs that remain stable despite heterogeneity. This property is particularly relevant for clinical translation, where biomarkers must perform across populations, platforms, and experimental protocols. At the same time, this robustness comes at the cost of reduced sensitivity to context-specific signals, underscoring the need for integrative analytical strategies that combine meta-analysis with stratified and single-cell approaches.

### 4.2. Additional Translational and Systems-Level Applications

Beyond biomarker discovery, transcriptomic meta-analysis has increasingly been used to address broader translational and systems-level questions in biomedical research. One emerging application involves identifying therapeutic candidates by comparing transcriptomic signatures. In this framework, disease-associated expression signatures derived from integrated datasets are contrasted with transcriptional responses induced by pharmacological compounds. Drugs capable of reversing disease-associated gene expression patterns can therefore be prioritized as potential therapeutic candidates. Large-scale perturbation resources, such as the Connectivity Map, have enabled the systematic implementation of this strategy, allowing researchers to identify compounds whose transcriptional effects oppose disease signatures derived from multiple independent studies [95,96]. A notable example is the study by Sirota et al. [97], who integrated multiple gene-expression datasets to construct a robust lung adenocarcinoma transcriptional signature and subsequently used drug-induced expression profiles to identify cimetidine as a candidate therapeutic agent, which was later confirmed in experimental models. Importantly, meta-analytic integration improves the robustness of the disease signature used in these comparisons, reducing the influence of cohort-specific transcriptional noise.

Another relevant application of transcriptomic meta-analysis lies in the identification of molecular disease subtypes. Many complex disorders that are clinically classified as single entities exhibit substantial transcriptional heterogeneity across patient populations. By integrating multiple independent transcriptomic datasets, meta-analysis can reveal stable gene-expression programs that distinguish biologically meaningful subgroups. This strategy has been particularly influential in oncology, where integrative transcriptomic analyses have enabled the definition of consensus molecular subtypes that better explain disease progression and therapeutic response than traditional clinical classifications [98]. Similar approaches have also been applied in immune-mediated diseases to identify transcriptional programs shared across pathological conditions or specific to particular therapeutic responses [11]. Through this lens, transcriptomic meta-analysis contributes not only to biomarker discovery but also to the molecular stratification of complex diseases.

### 4.3. Cross-Species Comparative and Evolutionary Applications

Beyond biomarker discovery and molecular stratification within a single disease context, transcriptomic meta-analysis also provides a powerful framework for identifying transcriptional programs that remain conserved across species [28,99]. This application is particularly valuable when the biological question cannot be addressed directly in humans or when the translational validity of animal models must be evaluated. In such cases, meta-analysis does not merely increase statistical power; rather, it filters gene-expression signals through an additional layer of biological reproducibility, prioritizing those responses that persist despite differences in taxonomy, tissue architecture, and experimental design. Consequently, cross-species integration can help distinguish core regulatory programs from lineage-specific transcriptional variation.

This comparative use of transcriptomic meta-analysis depends on the reliable mapping of homologous or orthologous genes across organisms, together with standardized preprocessing and effect integration strategies. The importance of this problem is reflected in the development of dedicated tools such as CoRMAP [47], which was specifically designed to retrieve and compare RNA-seq datasets from phylogenetically divergent species through orthogroup-based standardization. More broadly, cross-species transcriptomic integration frequently relies on orthology mapping resources such as HCOP, OrthoDB, and BioMart, which facilitate the identification and harmonization of homologous genes across organisms and improve the comparability of evolutionary and functional analyses.

By operating at the level of comparable gene sets rather than species-specific annotations, this framework enables the identification of conserved expression responses even when reference genomes, sequencing platforms, or analytical pipelines differ substantially.

In practice, cross-species transcriptomic meta-analysis has already proven useful in several biological contexts. Schall and Latham [100], for example, integrated RNA-seq datasets from multiple model species to examine the morula-to-blastocyst transition and showed that, although many differentially expressed genes were species-specific, a smaller subset of shared transcriptional changes converged on conserved metabolic and physiological pathways. Likewise, Farhadian et al. [101] combined transcriptomic data from wallaby, rat, and cow to identify a common gene signature associated with lactation, illustrating how meta-analysis can reveal conserved molecular programs underlying complex physiological traits. More recently, Beccacece et al. [102] analyzed 2144 publicly available samples from seven animal species and reported evolutionarily conserved transcriptional responses to PFAS exposure, including recurrent effects on lipid metabolism, immune processes, and hormone-related pathways. Together, these examples show that transcriptomic meta-analysis can support evolutionary inference, improve model-organism selection, and identify molecular responses that are robust not only across studies but across species boundaries.

From an interpretative perspective, this application is especially important because reproducibility across species imposes a stricter criterion than reproducibility across cohorts alone. A gene expression pattern repeatedly observed in independent datasets from different organisms is less likely to reflect a narrow experimental artifact and more likely to represent a biologically central response. At the same time, cross-species meta-analysis also exposes the limits of translational generalization by revealing which pathways are conserved and which remain species-dependent. Thus, rather than assuming that animal models faithfully reproduce human transcriptional states, this approach explicitly and quantitatively evaluates their molecular relevance.

## 5. Perspectives and Conclusions

Transcriptomic meta-analysis has emerged as a powerful strategy for extracting robust biological insights from the rapidly expanding landscape of gene expression studies. By integrating evidence across multiple independent experiments, this approach increases statistical power, mitigates study-specific biases, and prioritizes transcriptional signals that are reproducible across heterogeneous biological and technical contexts. In fields where individual transcriptomic studies are often limited by small sample sizes, variable designs, or platform-specific effects, meta-analysis provides a principled strategy for synthesizing fragmented evidence into coherent biological interpretation.

At the same time, integrating transcriptomic data across studies poses substantial methodological and conceptual challenges. Heterogeneity arising from experimental design, sequencing technology, biological resolution, and population structure is not an incidental complication but a defining feature of transcriptomic meta-analysis. As discussed throughout this review, effective integration requires explicit consideration of these sources of variability at every stage of the analytical pipeline, from dataset selection and preprocessing to normalization, batch-effect correction, and statistical modeling. Importantly, heterogeneity should not be treated solely as noise to be eliminated, but as information that constrains interpretation and informs the generalizability of meta-analytic findings.

Recent methodological advances have expanded the scope of transcriptomic meta-analysis beyond traditional gene-level differential expression. Network-based approaches, coexpression meta-analysis, and the integration of single-cell transcriptomic data now enable systems-level interpretations that capture regulatory programs, cellular states, and functional organization. These developments have strengthened the link between transcriptomic meta-analysis and systems biology, allowing reproducible molecular patterns to be interpreted in the context of pathways, cell-type composition, and metabolic processes. Nevertheless, the increasing complexity of transcriptomic data also demands careful methodological discipline to avoid overcorrection, loss of biological signal, or spurious convergence driven by technical artifacts.

From an applied perspective, transcriptomic meta-analysis has demonstrated clear value across a range of biomedical and biological contexts. Its contribution to biomarker discovery lies not in identifying universally altered genes, but in prioritizing transcriptional programs that remain stable across heterogeneous conditions. Similarly, its use in therapeutic prioritization, disease stratification, and cross-species comparative analysis highlights the importance of reproducibility as a criterion for translational relevance. At the same time, these applications underscore an inherent trade-off: increased robustness often comes at the expense of sensitivity to context-specific or rare biological signals, reinforcing the need for complementary approaches such as stratified analyses and single-cell resolution.

Looking forward, the continued expansion of public repositories, improved metadata standards, and FAIR-compliant data sharing will further strengthen transcriptomic meta-analysis. At the same time, advances in statistical modeling, machine learning, and multi-omics integration will support more flexible representations of heterogeneity and more nuanced biological inference. In this context, transcriptomic meta-analysis should be viewed not merely as a statistical procedure for combining datasets, but as a rigorous framework for reproducible biological inference across heterogeneous experimental systems.

In conclusion, transcriptomic meta-analysis provides a conceptual and methodological foundation for integrating heterogeneous gene expression data into coherent biological insight. When applied with careful attention to data quality, heterogeneity, and analytical assumptions, it enables the transformation of disparate transcriptomic studies into robust, interpretable, and biologically meaningful integrative evidence across experimental systems and scales.

## Figures and Tables

**Table 1:** Major transcriptomic technologies and their implications for meta-analysis. This table compares the principal transcriptomic technologies used in gene expression studies, including microarrays, bulk RNA sequencing, single-cell RNA sequencing, and spatial transcriptomics. For each approach, the measurement unit, biological resolution, main strengths, and key limitations relevant to meta-analysis are summarized. The comparison highlights how differences in data structure and resolution (from bulk tissue averages to single-cell and spatially resolved measurements) directly influence variability, interpretability, and cross-study integration strategies. In particular, while bulk approaches provide stable, widely available datasets, they are affected by compositional biases, whereas single-cell and spatial technologies offer higher biological resolution at the cost of increased sparsity, batch effects, and analytical complexity. These distinctions underscore that the choice of transcriptomic technology is a critical determinant of meta-analytic design and the interpretation of integrated gene expression signals. Abbreviations: RNA-seq, RNA sequencing.

Technology	Measurement Unit	Biological Resolution	Main Strengths	Key Limitations for Meta-Analysis
Microarrays	Probe-level signal	Bulk tissue	Large legacy datasets; stable variance	Platform-specific bias; limited dynamic range
Bulk RNA-seq	mRNA abundance	Bulk tissue	High sensitivity; wide dynamic range	Cell-type averaging; compositional effects
Single-cell RNA-seq	mRNA per cell	Cellular	Cell-type resolution; heterogeneity	Sparsity; strong batch effects; annotation variability
Spatial transcriptomics	mRNA + spatial coordinates	Cellular + spatial	Tissue context; functional localization	Limited resolution; platform heterogeneity; complex integration

**Table 2:** Public repositories providing transcriptomic data for integrative analysis. This table summarizes the principal public repositories used in transcriptomic meta-analysis, outlining their data types, biological scope, strengths, and key limitations affecting cross-study integration. Although resources such as GEO and SRA provide extensive and diverse datasets that enable large-scale reanalysis, their utility is often constrained by inconsistent metadata annotation and heterogeneous preprocessing. In contrast, more specialized repositories, including single-cell and spatial transcriptomic databases, offer higher-resolution data but introduce additional challenges related to standardization, annotation consistency, and methodological variability. These differences highlight that data availability alone does not guarantee suitability for integration. Instead, the analytical value of each repository depends on the extent to which its datasets support reproducible preprocessing, metadata harmonization, and biologically meaningful comparison across studies. Repository characteristics may vary depending on metadata completeness, preprocessing status, and dataset curation quality.

Repository	Data Types ^a^	Biological Scope	Key Strengths	Main Limitations for Meta-Analysis	Website
GEO (NCBI)	MA, RNA, SC	Multi-species, diverse tissues	Extensive legacy datasets; rich metadata for many studies	Variable annotation quality; heterogeneous preprocessing	www.ncbi.nlm.nih.gov/geo/ accessed on 18 May 2026
SRA (NCBI)	RNA, SC	Multi-species	Access to raw data enables full reprocessing	Minimal metadata; requires linkage to publications or GEO	www.ncbi.nlm.nih.gov/sra/ accessed on 18 May 2026
GTEx	RNA	Human tissues	High-quality, standardized tissue expression	Not disease-focused; limited experimental contrasts	www.gtexportal.org/home/ accessed on 18 May 2026
Single Cell Portal	SC	Multi-species, cell-type resolved	Specialized for single-cell data	Heterogeneous annotation across studies	https://singlecell.broadinstitute.org/single_cell accessed on 18 May 2026
SpatialDB/STOmicsDB	ST	Tissue-level spatial context	Preserves the spatial organization of expression	Limited standardization; emerging methodologies	www.spatialomics.org/SpatialDB/ accessed on 18 May 2026

^a^ Data type: MA, microarrays; RNA, RNA-seq; SC, scRNA-seq; ST, spatial transcriptomics.

**Table 3:** Comparison of seven major statistical approaches for transcriptomic meta-analysis. Fixed- and random-effects models constitute classical meta-analytic frameworks, with random-effects models generally preferred for biological data due to expected heterogeneity. *p*-value combination methods (Fisher’s and Stouffer’s) are useful when effect sizes are unavailable, but they lose information about effect magnitude. Effect-size aggregation yields interpretable effect estimates and supports heterogeneity assessment. Pathway-level approaches increase power by aggregating gene-level evidence within biological pathways. Bayesian methods offer flexible probabilistic frameworks that incorporate prior knowledge. Method selection should consider data availability (*p*-values vs. effect sizes), expected heterogeneity, computational resources, and whether effect magnitude or biological interpretation is the primary goal.

Method	Statistical Assumptions	Key Strengths	Key Weaknesses
**Fixed Effects Model**	All studies estimate the same true effect Between-study variation is zero Differences are due to sampling error only Homogeneous study populations	Simple, intuitive interpretation Maximum statistical power when assumptions hold Precise estimates with narrow confidence intervals Computationally efficient	Unrealistic homogeneity assumption for most biological data Underestimates uncertainty when heterogeneity exists Overly narrow confidence intervals High false positive rate with heterogeneity
**Random Effects Model**	True effects vary across studies Study-specific effects are drawn from a normal distribution Accounts for both within-study and between-study variance Heterogeneity is real, not just sampling error	Realistic for most biological meta-analyses Explicitly accounts for heterogeneity More conservative, with appropriate confidence intervals Generalizable to broader populations Robust to study differences	Requires sufficient studies (≥5–10) for reliable heterogeneity estimation Less statistical power than fixed effects Wider confidence intervals Sensitive to the heterogeneity estimation method May be unstable with a few studies
**Fisher’s Method (*p*-value combination)**	*p*-values are uniformly distributed under the null hypothesis Studies are independent Test statistics follow a chi-square distribution No effect size information required	Does not require effect sizes or standard errors Works with diverse study designs Simple to implement Handles missing data well Platform-agnostic	Loses information by reducing to *p*-values Cannot estimate effect magnitude or direction Sensitive to small *p*-values (can be dominated by a single study) No heterogeneity assessment Assumes independence
**Stouffer’s Method (Z-score combination)**	Z-scores are normally distributed under the null Studies are independent Can incorporate study weights Assumes symmetric distributions	Allows for study weighting (e.g., by sample size) More balanced than Fisher’s method Less sensitive to extreme *p*-values Computationally efficient Provides a combined Z-score	Still loses effect size information Requires *p*-values or Z-scores Cannot estimate effect magnitude The weighting scheme affects the results No direct heterogeneity measure
**Effect-Size Aggregation (Cohen’s d/Hedges’ g)**	Effect sizes are normally distributed Within-study variances are known or estimable Studies measure comparable outcomes Standardized mean differences are appropriate	Provides interpretable effect magnitude Allows heterogeneity assessment (I^2^, τ^2^) Enables meta-regression and subgroup analysis Standard errors quantify uncertainty Forest plots for visualization	Requires raw data or summary statistics Assumes comparable effect definitions Sensitive to outliers Requires careful standardization May be biased with small samples (use Hedges’ g correction)
**Pathway-Level Meta-Analysis (GSEMA)**	Gene sets represent biological pathways Pathway effects aggregate gene-level evidence Accounts for gene-gene correlations Pathway databases are accurate and current	Increases statistical power through aggregation Biologically interpretable results Reduces the multiple testing burden Captures coordinated gene expression More robust to individual-gene noise	Depends on the quality and completeness of pathway databases May miss novel biology outside known pathways Pathway definitions vary across databases Computationally intensive Requires careful pathway selection
**Bayesian Meta-Analysis**	Prior distributions specified for parameters Likelihood function for observed data Posterior distributions via Bayes’ theorem Hierarchical modeling of study effects	Incorporates prior knowledge explicitly Provides full posterior distributions Natural handling of uncertainty Flexible modeling of complex structures Probabilistic interpretation Handles small sample sizes well	Requires prior specification (can be subjective) Computationally intensive (MCMC sampling) Requires specialized expertise Sensitive to prior choice with limited data Longer computation time

**Table 4:** Comparison of computational tools for transcriptomic meta-analysis. spanning R packages, Python tools, and web-based platforms. R packages (MetaDE, ImaGEO, GEDI, DExMA, reanalyzerGSE) offer flexibility and integration with the R/Bioconductor ecosystem but require programming expertise. Web-based platforms (MAGE, ExpressAnalyst) provide user-friendly interfaces suitable for researchers without programming skills, but have limitations in data size and customization. Specialized tools focus on specific aspects: pyComBat for Python-based batch correction, MetaPath/MetaOmics for pathway-level analysis, and SelectBCM for batch correction method selection. Tool selection should consider programming expertise, data size, analysis complexity, reproducibility requirements, and whether an integrated workflow or modular approach is preferred. For most researchers, ImaGEO or GEDI provides a good starting point for R-based workflows, while ExpressAnalyst suits those who prefer web interfaces.

Tool	Main Features	Key Limitations	Recommended For
**MetaDE**	R package Multiple meta-analysis methods (Fisher, Stouffer, AW-Fisher, maxP, minP, rOP, rth-ordered *p*-value) Handles both microarray and RNA-seq FDR control and permutation testing Supports heterogeneous platforms Visualization functions	Limited to *p*-value combination approaches No effect-size meta-analysis Requires preprocessed data Limited batch correction capabilities Documentation could be more comprehensive	P-value combination meta-analysis Cross-platform integration Large-scale gene screening Comparing multiple combination methods Users comfortable with R
**ImaGEO**	R package Integrated workflow from GEO data retrieval to meta-analysis Automated data download and preprocessing Multiple normalization methods Batch effect correction (ComBat) Effect-size and *p*-value meta-analysis Comprehensive visualization	Primarily designed for GEO data May struggle with very large datasets Limited customization of preprocessing Requires an internet connection for GEO access Memory intensive	GEO-based meta-analyses End-to-end workflow automation Users wanting an integrated pipeline Moderate-scale studies Researchers new to meta-analysis
**GEDI**	R package Comprehensive multi-platform integration Advanced batch correction methods Quality control and diagnostic tools Supports microarray and RNA-seq Flexible preprocessing pipelines Modern implementation with updated methods	Relatively new tool (limited user base) Documentation still developing May have a learning curve Computational requirements for large datasets Limited long-term validation	Modern multi-platform integration Users wanting the latest methods Complex preprocessing needs Quality control emphasis Researchers are comfortable with R and new tools
**DExMA**	R package (Bioconductor) Differential expression meta-analysis Multiple statistical methods Pathway enrichment integration Handles missing genes across platforms Meta-regression capabilities Comprehensive documentation	Steeper learning curve Requires careful data formatting Computational intensity with large studies Limited real-time analysis capabilities May require Bioconductor expertise	Differential expression focus Pathway-level analysis Meta-regression analyses Users familiar with Bioconductor Complex study designs
**MAGE**	Web-based platform User-friendly web interface No programming required Automated preprocessing Multiple meta-analysis methods Interactive visualizations Export results in multiple formats	Limited to web interface capabilities Data size restrictions Less flexibility than command-line tools Internet connection required Privacy concerns with sensitive data Limited customization	Users without programming experience Small to moderate datasets Quick exploratory analyses Teaching and demonstrations Non-sensitive public data
**ExpressAnalyst**	Web-based platform Comprehensive transcriptomics workflow Data upload, normalization, batch correction Statistical analysis and visualization Pathway enrichment and network analysis User-friendly interface Extensive documentation and tutorials	Web-based limitations (data size, computation time) Less control than command-line tools Requires an internet connection May not support all specialized analyses Data privacy considerations	End-to-end transcriptomics analysis Users preferring web interfaces Educational purposes Rapid exploratory analysis Researchers without bioinformatics expertise
**reanalyzerGSE**	R package Reproducible GEO data reanalysis Automated pipeline for meta-analysis Emphasis on reproducibility and documentation FAIR principles integration Version control and provenance tracking Standardized reporting	Focused on GEO data specifically It may be overly structured for exploratory work Learning curve for reproducibility features Requires R and version control familiarity Documentation-heavy (pro and con)	Reproducible research emphasis GEO-based systematic reviews Publication-quality analyses FAIR data principles compliance Collaborative projects requiring documentation
**MetaPath/MetaOmics**	Pathway-level meta-analysis R package Integration with pathway databases (KEGG, Reactome, GO) Gene set enrichment approaches Handles gene-level and pathway-level data Visualization of pathway results	Depends on the pathway database quality May miss novel biology Computational intensity for large pathway sets Requires careful pathway selection Limited to organisms with good pathway annotations	Pathway-level hypotheses Biological interpretation focus Weak individual gene signals Reducing the multiple testing burden Well-annotated organisms (human, mouse)
**pyComBat**	Python package Python implementation of ComBat batch correction Handles microarray and RNA-seq data Parametric and non-parametric modes Integration with the Python data science ecosystem Efficient implementation	Focused solely on batch correction (not full meta-analysis) Requires a Python environment Less comprehensive than R ComBat implementations Limited additional preprocessing features Requires integration with other tools for a complete workflow	Python-based workflows Batch correction as a standalone step Integration with Python ML/AI pipelines Users preferring Python over R Computational biology in the Python ecosystem
**SelectBCM**	R package/tool Automated batch correction method selection Compares multiple batch correction approaches Dataset-specific recommendations Performance evaluation metrics Guides optimal method choice	Relatively specialized tool Adds computational overhead (testing multiple methods) May not always identify a clear winner Requires understanding of evaluation metrics Limited to the batch correction step	Uncertain which batch correction method to use Comparing batch correction approaches Dataset-specific optimization Methodological research Users want a data-driven method selection

**Table 5:** Major normalization and batch correction methods for transcriptomic meta-analysis. Normalization methods (Quantile, TMM, VST) address within-platform technical variation and are typically applied before batch correction. Batch correction methods (ComBat, reComBat, Rank-In, RUV, PEER) address between-study or between-batch variation, which is essential for meta-analysis. ComBat remains the most widely used batch correction method due to its effectiveness and broad applicability, though reComBat offers advantages for incremental data addition. RUV and PEER provide alternatives when control genes or samples are available, or when large sample sizes permit factor-based approaches. Rank-In offers a distribution-free approach for extreme heterogeneity but loses absolute expression information. Method selection should consider platform type (microarray vs. RNA-seq), data characteristics (sample size, heterogeneity), availability of batch labels or control genes, and whether absolute expression levels or relative rankings are more important for downstream analysis. For most meta-analyses, quantile normalization (microarray) or TMM/VST (RNA-seq) followed by ComBat batch correction provides a robust starting point.

Method	Platform Compatibility	When to Use	Key Limitations
**Quantile Normalization**	Affymetrix arrays Illumina arrays RNA-seq (with caution) Cross-platform integration	Assumes similar overall expression distributions across samples Technical variation dominates biological variation Large sample sizes Homogeneous sample types	Primarily microarray Forces identical distributions (may distort true biological differences) Not appropriate when samples are expected to differ substantially Can introduce artifacts in RNA-seq data May mask true biological heterogeneity Assumes most genes are not differentially expressed
**TMM (Trimmed Mean of M-values)**	RNA-seq count data Any sequencing platform Platform-agnostic for RNA-seq	RNA-seq normalization Accounts for library size and composition Differential expression analysis Most genes are not differentially expressed	RNA-seq only Assumes most genes are not differentially expressed May fail with extreme composition bias Requires count data (not applicable to microarray) Not suitable for highly unbalanced differential expression
**VST (Variance Stabilizing Transformation)**	RNA-seq count data DESeq2 workflow Any RNA-seq platform	RNA-seq data with count-based analysis Stabilizing variance across the expression range Visualization and clustering Downstream analyses requiring homoscedasticity	RNA-seq only Specific to the DESeq2 framework Requires count data Not applicable to microarray May not preserve exact fold changes Computationally intensive for very large datasets
**ComBat**	Microarray (any platform) RNA-seq (log-transformed) Cross-platform integration Multi-batch designs	Known batch effects present Multiple batches/studies to integrate Batch variable known and documented Sufficient samples per batch	Requires known batch labels May over-correct and remove true biological variation Assumes batch effects follow a specific parametric model Can introduce artifacts if batches are confounded with biology Requires adequate samples per batch (≥3–5)
**reComBat (2022)**	Microarray RNA-seq Cross-platform Reference-based correction	Reference dataset available New data to be integrated with existing corrected data Incremental data addition Maintaining consistency with previous corrections	Requires an appropriate reference dataset Reference quality critical May propagate reference biases More complex than standard ComBat Limited validation in diverse contexts
**Rank-In (Rank-based Inverse Normal transformation)**	Microarray RNA-seq Cross-platform integration Heterogeneous data types	Extreme heterogeneity across platforms Distribution-free approach needed Cross-platform meta-analysis When the parametric assumptions are questionable	Loses absolute expression information (converts to ranks) May reduce statistical power Not suitable when the absolute expression levels are important Interpretation less intuitive May mask true effect sizes
**pyComBat**	Microarray RNA-seq Python-based workflows Integration with ML pipelines	Python-based analysis pipelines Integration with scikit-learn, pandas, and NumPy Machine learning workflows Users preferring the Python ecosystem	Python-specific (not R) Less mature than R ComBat Smaller user community Limited integration with specialized bioinformatics tools Documentation is less comprehensive than the R version
**RUV (Remove Unwanted Variation)**	RNA-seq Microarray Single-cell RNA-seq Any platform with control genes/samples	Control genes or samples available Unwanted technical variation present Batch effects without clear batch labels Negative control genes identified	Primarily RNA-seq Requires control genes or samples (not always available) Control selection is critical and can be challenging Computationally intensive Multiple RUV variants (RUVg, RUVs, RUVr) with different requirements May require iteration to optimize
**PEER (Probabilistic Estimation of Expression Residuals)**	RNA-seq Microarray eQTL studies Large-scale expression studies	Large sample sizes (>100 samples) Unknown confounders present eQTL mapping Factor analysis approach desired	Primarily RNA-seq large sample sizes for stability Computationally intensive The number of factors to remove must be specified May remove the true biological signal if over-applied Black-box approach (factors not always interpretable)

## Data Availability

No new data were created or analyzed in this study. Data sharing is not applicable to this article.
